# Decreased mitochondrial NAD+ in WRN deficient cells links to dysfunctional proliferation

**DOI:** 10.18632/aging.206236

**Published:** 2025-04-02

**Authors:** Sofie Lautrup, Shi-Qi Zhang, Shinichiro Funayama, Lisa Lirussi, Tina Visnovska, Hoi-Hung Cheung, Marc Niere, Yuyao Tian, Hilde Loge Nilsen, Geir Selbæk, Janna Saarela, Yoshiro Maezawa, Koutaro Yokote, Per Nilsson, Wai-Yee Chan, Hisaya Kato, Mathias Ziegler, Vilhelm A. Bohr, Evandro F. Fang

**Affiliations:** 1 Department of Clinical Molecular Biology, University of Oslo and Akershus University Hospital, Lørenskog 1478, Norway; 2 Department of Endocrinology, Hematology and Gerontology, Chiba University Graduate School of Medicine, Chiba 260-0856, Japan; 3 Department of Diabetes, Metabolism and Endocrinology, Chiba University Hospital, Chiba 260-8677, Japan; 4 Department of Microbiology, Oslo University Hospital, Oslo 0450, Norway; 5 School of Biomedical Sciences, Faculty of Medicine, The Chinese University of Hong Kong, Shatin, N.T., Hong Kong; 6 Department of Biomedicine, University of Bergen, Bergen 5009, Norway; 7 Genetics and Aging Research Unit, McCance Center for Brain Health, Mass General Institute for Neurodegenerative Disease, Department of Neurology, Massachusetts General Hospital, Charlestown, MA 02129, USA; 8 Institute of Clinical Medicine, University of Oslo, Oslo 0372, Norway; 9 The Norwegian National Centre for Aging and Health, Vestfold Hospital Trust, Tønsberg 3103, Norway; 10 Department of Geriatric Medicine, Oslo University Hospital, Oslo 0450, Norway; 11 Centre for Molecular Medicine Norway (NCMM), University of Oslo, Oslo 0372, Norway; 12 Institute for Molecular Medicine Finland (FIMM), HiLIFE, University of Helsinki, Helsinki, Finland; 13 Department of Medical Genetics, Oslo University Hospital, Oslo 0450, Norway; 14 Department of Neurobiology, Care Sciences and Society, Center for Alzheimer Research, Division of Neurogeriatrics, Karolinska Institutet, Solna 17164, Sweden; 15 Leibniz Institute for Natural Product Research and Infection Biology, Hans Knöll Institute, Jena 07745, Germany; 16 Department of Cellular and Molecular Medicine, Center for Healthy Aging, University of Copenhagen, Copenhagen 1172, Denmark; 17 The Norwegian Centre on Healthy Ageing (NO-Age) and The Norwegian National Anti-Alzheimer’s Disease (NO-AD) Networks, Oslo 0372, Norway

**Keywords:** Werner syndrome, premature aging, NAD+, mitochondria, proliferation

## Abstract

Werner syndrome (WS), caused by mutations in the RecQ helicase WERNER (*WRN*) gene, is a classical accelerated aging disease with patients suffering from several metabolic dysfunctions without a cure. While, as we previously reported, depleted NAD^+^ causes accumulation of damaged mitochondria, leading to compromised metabolism, how mitochondrial NAD^+^ changes in WS and the impact on WS pathologies were unknown. We show that loss of WRN increases senescence in mesenchymal stem cells (MSCs) likely related to dysregulation of metabolic and aging pathways. In line with this, NAD^+^ augmentation, via supplementation with nicotinamide riboside, reduces senescence and improves mitochondrial metabolic profiles in MSCs with *WRN* knockout (*WRN^−/−^*) and in primary fibroblasts derived from WS patients compared to controls. Moreover, *WRN* deficiency results in decreased mitochondrial NAD^+^ (measured indirectly via mitochondrially-expressed PARP activity), and altered expression of key salvage pathway enzymes, including NMNAT1 and NAMPT; ChIP-seq data analysis unveils a potential co-regulatory axis between WRN and the NMNATs, likely important for chromatin stability and DNA metabolism. However, restoration of mitochondrial or cellular NAD^+^ is not sufficient to reinstall cellular proliferation in immortalized cells with siRNA-mediated knockdown of *WRN*, highlighting an indispensable role of WRN in proliferation even in an NAD^+^ affluent environment. Further cell and animal studies are needed to deepen our understanding of the underlying mechanisms, facilitating related drug development.

## INTRODUCTION

The accelerated aging disease Werner syndrome (WS) is a segmental progeria caused by mutations in the gene encoding the RecQ DNA helicase WERNER (WRN) protein [1]. WS is an autosomal recessive-inherited disease and its estimated global incidence ranges from 1 in 1 million to 1 in 10 million births, with a higher incidence in Japan [2]. The first sign of WS is lack of growth spurt in the early teens, followed by more obvious aging-related characteristics such as cataracts, skin atrophy, greying and loss of hair, wrinkles, loss of fat, and atherosclerosis developing at the age of 20 to 30 years [3]. WS patients also experience severe metabolic dysfunctions including diabetes, fatty liver, and dyslipidemia [3–5]. At the cellular level, loss of functional WRN results in compromised DNA repair, telomere attrition, premature senescence, cell cycle defects and compromised mitophagy [4, 6–9]. Moreover, we recently showed that loss of WRN in stem cells leads to accelerated adipogenesis and results in dysfunctional adipocyte metabolism [5].

Nicotinamide adenine dinucleotide (NAD^+^) is an essential metabolite involved in multiple processes including redox homeostasis, the regulation of cellular metabolism, energy transduction, DNA repair, mitophagy, inflammation, and neuronal function [10, 11]. Additionally, decreased NAD^+^ is associated with aging and accelerated aging across multiple tissues and organisms [6, 11–15]. NAD^+^ is highly compartmentalized with the major pools of NAD^+^ being nuclear, cytoplasmic, and mitochondrial [16–19]. NAD^+^ is likely transported from the cytoplasm to other subcellular organelles, including the mitochondria, via SLC25A51 [20, 21]. Moreover, proteins involved in both biosynthesis and consumption of NAD^+^ contribute to the sub-cellular regulation of NAD^+^ metabolism. As an example, the three-mammalian nicotinamide mononucleotide adenylyl transferases (NMNAT1-3), converting nicotinamide mononucleotide (NMN) to NAD^+^, show different subcellular localization. NMNAT1 is mostly expressed in the nucleus, and NMNAT2 associated with the Golgi membrane, whereas NMNAT3 is suggested to be primarily expressed within the mitochondria [22–24]. Despite extensive research, very little is known about the effects of aging on the subcellular regulation of NAD^+^ metabolism [25].

Our previous work demonstrated that NAD^+^ depletion is an essential factor in the accelerated aging features of WS models (patient derived plasma, cells, *Caenorhabditis elegans*, *Drosophila melanogaster*) at least partially via inhibition of mitophagy leading to accumulation of dysfunctional mitochondria [6]. Moreover, we showed that stem cell proliferation in a *Drosophila* model of WS was rescued by NAD^+^ augmentation [6], and that NAD^+^ augmentation rescued accelerated adipocyte metabolism in both stem cells and zebrafish (*Danio rerio*) [5]. To further elucidate the mechanisms of NAD^+^ dysregulation in WS and the effect on one main feature of WS, namely loss of cell proliferation, we examined the subcellular regulation of NAD^+^ metabolism in cellular models of WS in relation to proliferation. Our results indicated compromised NAD^+^ metabolism in WS while NAD^+^ augmentation decreased senescence in both WS mesenchymal stem cells (MSCs) and primary fibroblasts, shedding light on potential therapeutics.

## RESULTS

### Loss of WRN in mesenchymal stem cells disrupts cellular proliferation and metabolic pathways

**Figure 1:**
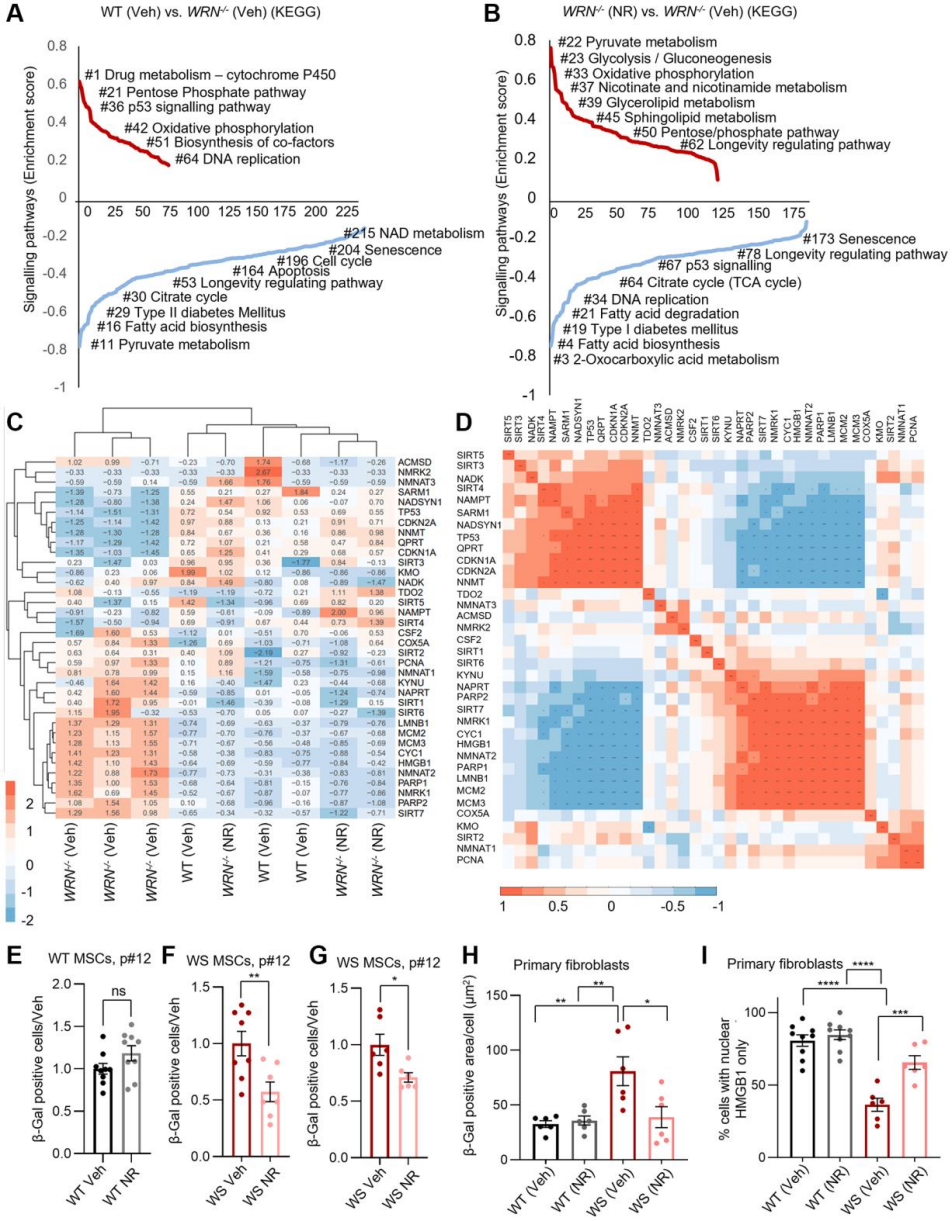
**WRN is essential in maintaining metabolic, mitochondrial, and other longevity-related pathways as well as in inhibiting senescence in mesenchymal stem cells.** (**A**, **B**) Gene-set-enrichment analysis demonstrates upregulated and downregulated signaling pathways (KEGG pathways) in WT and *WRN^−/−^* MSCs with/without 1 mM NR treatment for 24 h. The KEGG terms were ranked on the basis of enrichment scores. The upregulated KEGG pathways and downregulated KEGG pathways are summarised separately. (**C**) Heat map data showing fpkm changes of significantly up- or down-regulated genes (DEGs) related to NAD^+^ metabolism in the comparisons shown (list of target genes is found in Supplementary Table 1; full list of fpkm values is found in Supplementary File 1). *WRN^−/−^* (Veh) clusters alone, whereas *WRN^−/−^* (NR) and WT (Veh) cluster together. (**D**) Correlation matrix of fpkm values showing the correlation between expression of NAD^+^ related DEGs and proliferation/senescence related markers. (**E**–**G**) Senescence evaluation by senescence associated β-Galactosidase (SA-β-Gal) staining of MSCs derived from control (WT) and WS patients (WS). SA-β-Gal positive cells decreased with 11–18 days of 1 mM NR treatment in WRN^*−/−*^ MSCs (Student’s *t*-test, *p*-values = 0.0097 and 0.0156, respectively) (**F**, **G**), but not in WT MSCs (Student’s *t*-test, *p*-value = 0.2029) (**E**). SA-β-Gal positive MSCs relative to total number of cells were quantified from two biological experiments per cell line. (**H**) SA-β-Gal staining of primary fibroblasts from healthy control donors (WT) and WS patients (WS). SA-β-Gal staining was increased in WS patient derived fibroblasts compared to healthy controls (WT) (Two-way ANOVA, Tukey’s multiple comparisons test, *p*-value = 0.0036). SA-β-Gal staining decreased with 10 days 1 mM NR treatment in WS patient derived primary fibroblasts (Two-way ANOVA, Tukey’s multiple comparisons test, *p*-value = 0.0115) (**F**, **G**), but not in WT MSCs (Two-way ANOVA, Tukey’s multiple comparisons test, *p*-value = 0.9930) (Senescence evaluation by Spider β-Gal (Dojindo) staining of primary fibroblasts from healthy donors (WT) or WS patients (WS) without or with 1 mM NR for 10 days prior to staining are found in Supplementary Figure 1). (**I**) Staining of HMGB1 in primary fibroblasts from healthy donors (WT) or WS patients (WS) without or with 1 mM NR treatment for 10 days prior to staining. The number of cells with nuclear HMGB1 only relative to the total number of cells is shown in the figure from three biological repeats from two WT cell lines and two WS cell line. The percentage of cells with nuclear HMGB1 was decreased in WS-derived primary fibroblasts compared to WT (Two-way ANOVA, Tukey’s multiple comparisons test, *p*-value < 0.0001). Supporting the SA-β-Gal staining, 10 days 1 mM NR treatment increased the proportion of WS-derived fibroblasts with nuclear HMGB1 staining compared to vehicle treated cells significantly (Two-way ANOVA, Tukey’s multiple comparisons test, *p*-value = 0.0006), indicating decreased senescence with NR treatment.

The helicase WRN has an essential role in DNA repair and genomic maintenance, as evidenced by the disruption of cellular proliferation, accelerated senescence, and stem cell dysfunctions seen in primary cells from WS patients [2–5, 7–9, 26]. Additionally, metabolic abnormalities are common in WS patients, where mitochondrial dysfunction and compromised mitophagy play important roles [5, 6]. Using our recently published RNA sequencing data from MSCs with a constituent knock out of *WRN* (*WRN*^−/−^) and an isogenic control [5], we evaluated significantly altered pathways between WT and *WRN*^−/−^ (Figure 1A). Multiple pathways related to cellular metabolism and mitochondrial function changed in *WRN*^−/−^ cells compared to WT cells, including the pentose phosphate pathway, oxidative phosphorylation, and pyruvate metabolism (Figure 1A). Also, proliferation related pathways were disrupted upon *WRN* deficiency. Interestingly, only 24 h treatment with 1 mM nicotinamide riboside (NR), an NAD^+^ precursor, rescued multiple pathways in the *WRN*^−/−^ cells, including increased expression of genes driving mitochondrial and metabolism-related pathways, as well as proliferation-related pathways (Figure 1B). This indicated that NR treatment targets mitochondria in *WRN*^−/−^ MSCs. The rescue of proliferation-related pathways supports our previous findings showing complete restoration of stem cell proliferation following NR or NMN treatment in a *Drosophila* model of WS, with the mechanism unknown [6].

Heatmap analysis of differentially expressed genes related to proliferation and NAD^+^ metabolism using fpkm (fragments Per Kilobase of transcript per Million mapped reads) values with a cut off at fold-change > 1.5 and *p*-value < 0.05 analyzed with DESeq2, showed a clustering of the *WRN*^−/−^ cells (WRN^−/−^ (Veh)), compared to another clustering of WT together with *WRN*^−/−^ with NR treatment (WT (Veh), WRN^−/−^ (NR)) (Figure 1C). This suggested that NR treatment of *WRN*^−/−^ cells at least partially restored the expression of this selection of genes to WT levels (Figure 1C). Using a correlation matrix, we saw that the expression of NAD^+^ related genes such as the rate limiting enzyme in the Salvage pathway, Nicotinamide phosphoribosyl transferase (*NAMPT*), which recycles nicotinamide (NAM) to NMN, and NAD Synthetase 1 (*NADSYN1*), which catalyzes the conversion of nicotinic acid adenine dinucleotide (*NAAD*) to NAD^+^, the final step in the *de novo* pathway correlated positively with one another, but negatively to both *NMNAT1*, *NMNAT2*, *QRPT* (Kynurenine pathway) and *NNMT (Salvage pathway)*. *NMNAT1* correlated positively with *SIRT2*, *COX5A* and *NADK* (not significant after adjusted *p*-value), while *NMNAT2* correlated positively with *SIRT7*, *NAPRT*, *PARP1*, and *NMRK1*, all important in NAD^+^ biosynthesis (Figure 1D and Supplementary Table 1).

Due to premature senescent features of WS cells, and the essential role of WRN in DNA repair, we examined the correlation between NAD^+^ related genes and senescence/proliferation associated genes. We observed a positive correlation between *NAMPT* and *NADSYN1* to pro-senescence genes including *CDKN1A* and *CDKN2A*, and a negative correlation with *LMNB1*, *MCM2*, and *MCM3*, previously shown to be downregulated during senescence and proliferative arrest [27, 28].

On the other hand, we observed a negative correlation between the *NMNAT1-2* and proliferation-associated genes such as *TP53*, *CDKN1A*, and *CDKN2A* known to be upregulated during senescence [29, 30] (Figure 1D). Moreover, the *NMNAT1-2* correlated positively with *LMNB1*, *MCM2*, and *MCM3* previously shown to be downregulation during senescence and proliferative arrest [27, 28]. Opposite, to the other two NMNATs, *NMNAT3* did not show any significant correlations with adjusted *p*-value. However, the pattern of *NMNAT3* correlations were opposite to that of the other two *NMNATs*, resembling the patterns of *NAMPT* and *NADSYN1*. Combined, these results suggest a regulatory link between NAD^+^ related genes and proliferation.

To validate the results from the RNA sequencing analysis, we evaluated the effect of NR treatment on senescence markers in the WT and *WRN*^−/−^ MSCs and in primary fibroblasts derived from healthy control donors and WS patients (Supplementary Table 2) [9]. In line with the RNA seq results, we observed decreased senescence, measured with senescence associated β-Galactosidase (SA-β-Gal) staining, in *WRN*^−/−^ MSCs at passage 12 after both 11 and 18 days with 1 mM NR treatment (Figure 1F, 1G and Supplementary Figure 1A–1F). No change was observed in WT MSCs with NR treatment (Figure 1E and Supplementary Figure 1E, 1F). An increase SA-β-Gal staining and a decreased nuclear localization of HMGB1 indicating accelerated senescence was observed in WS patients-derived fibroblasts compared to WT fibroblasts. Interestingly, 10 days treatment with 1 mM NR decreased the senescence markers, as indicated by significantly decreased β-Gal positive area in WS fibroblasts and a significant increased percentage of cells with nuclear HMGB1 localization compared to WS Veh (Figure 1H, 1I and Supplementary Figure 1G–1K). Neither in the primary fibroblasts was a difference observed in WT cells treated with NR compared to vehicle (Figure 1H, 1I and Supplementary Figure 1G–1K). Combined with previous findings [1, 2, 6, 26], these data suggest that WRN is essential in proliferation and senescence inhibition, while NAD^+^ replenishment diminished senescence in MSCs and primary fibroblasts.

### NAD^+^ synthesis-related proteins are dysregulated by *WRN*-KD in HEK293 cells

**Figure 2:**
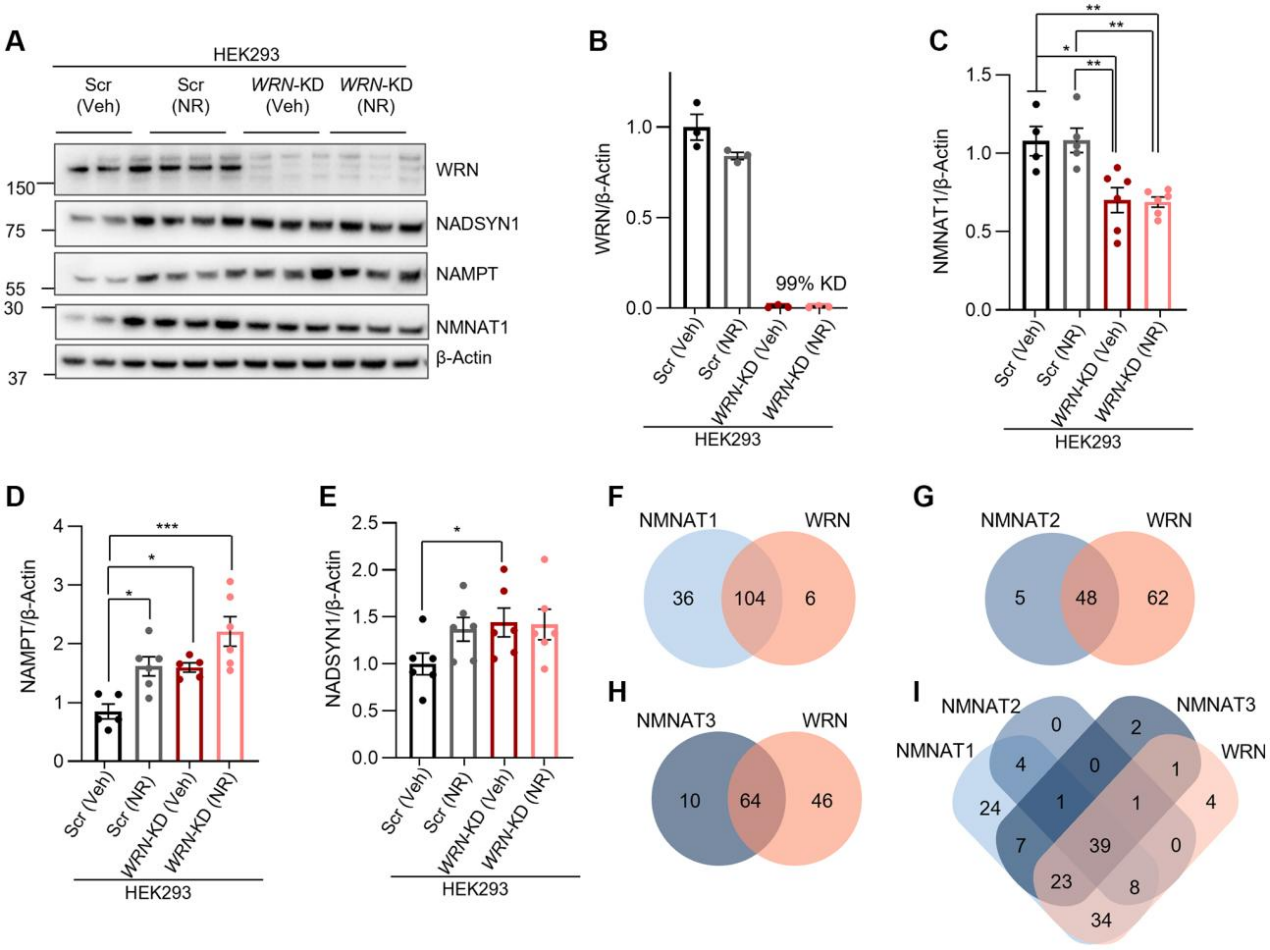
**WRN regulates main NAD^+^ synthetic proteins and shares many transcription targets with NMNAT1-3.** (**A**) Representative western blots of RIPA extracts from HEK293 parental cells with either 30 nM Scramble siRNA (Scr) or 30 nM WRN siRNA (*WRN*-KD) followed by 24 h 1 mM NR treatment*.* (**B**–**F**) Quantification of western blots from 4–6 biological repeats. Statistical analysis was performed in GraphPad Prism with either One-way ANOVA with Šidák multiple comparison’s test or Student’s *t*-test. (**C**) NMNAT1 was significantly decreased in *WRN*-KD cells compared to Scr (Veh) and Scr (NR) (One-way ANOVA, Tukey’s multiple comparisons test, *p*-value = 0.0108, student’s *t*-test *p*-value = 0.0163), (**D**) NAMPT was significantly increased in *WRN*-KD (Veh) cells compared to Scr (Veh) (One-way ANOVA, Tukey’s multiple comparisons test, *p*-value = 0.0492, Student’s *t*-test, *p*-value = 0.0010) and (**E**) NADSYN1 was upregulated in *WRN*-KD (Veh) compared to Scr (Veh) (Student’s *t*-test, *p*-value = 0.0442). (**F**–**I**) Venn diagram showing the shared transcription targets of WRN and NMNAT1-3 based on the ENCODE transcription database.

Via the salvage pathway, the NAD^+^ precursor NR is first converted to NMN which is further catabolized to NAD^+^; the latter step is mediated by the central proteins NMNAT1, NMNAT2 or NMNAT3, depending on subcellular localization [25]. We evaluated the change in the expression level of both NMNAT1 and other proteins involved in NAD^+^ metabolism in HEK293 cells treated with siRNA targeting *WRN* (*WRN*-KD). First, we confirmed the knockdown efficiency of *WRN* to be 99% compared to Scramble siRNA (Scr) with western blotting (Figure 2A, 2B). Next, the expression level of NMNAT1 was found to be significantly lower in *WRN*-KD (Veh) HEK293 cells compared to Scr (Veh) (Figure 2A, 2C) supporting our previous findings in primary fibroblasts [6]. We also tested the detection of NMNAT2 and NMNAT3, but the commercially available antibodies tested did not show bands on the expected sizes or did not work (not shown). The protein levels of NAMPT and NADSYN1 were significantly increased in *WRN*-KD (Veh) cells compared to Scr (Veh) (Figure 2A, 2D, 2E). To increase intracellular NAD^+^ [6, 18], we treated the cells with 1 mM NR for 24 h prior to protein extraction. Interestingly, we did not see an effect of NR treatment on the expression of either NMNAT1 or NADSYN1 in Scr or *WRN*-KD cells. The expression of NAMPT was significantly increased in Scr with NR treatment compared to Scr (Veh) (One-way ANOVA, Tukey’s multiple comparisons test, *p*-value = 0.0325) (Figure 2A, 2D). There was a tendency towards increased expression of NAMPT in *WRN*-KD cells with NR compared to *WRN*-KD (Veh) (One-way ANOVA, Tukey’s multiple comparisons test, *p*-value = 0.1048) (Figure 2A, 2D).

Based on our findings of decreased protein expression of NMNAT1 in *WRN-*KD cells, we wondered whether the regulation of WRN and all the NMNATs were connected. Due to the participation of both WRN and the NMNATs in transcriptional regulation [26, 31], we explored ChIP sequencing datasets from the ENCODE Transcription factor target database for potential co-regulation by comparing transcription factors known to bind to specific gene promoters, as we described previously [6]. Our analysis showed that WRN has 110 targets, NMNAT1 has 140 targets, NMNAT2 has 53 targets, and NMNAT3 has 74 targets (lists of all targets in Supplementary File 2). We previously showed that NMNAT1 and WRN share most of their known targets including SIRT6, RAD21, BACH1 and more [6] (Figure 2F). Additionally, more than 40% of the targets are shared between NMNAT2 and WRN (Figure 2G), and more than 50% are shared between NMNAT3 and WRN (Figure 2H). Interestingly, 39 targets are shared between WRN and all three NMNATs (Figure 2I). The shared targets involved chromatin regulators (CHD1, CHD2, RAD21, CTCF, CHD7SMC3, GTF2F1), transcriptional regulators (SIN3A, REST, YY1, ZNF143, BACH1, JUND, TBP), and histone modifiers (KDM4A, KDM5B, EP300, H2AFZ, HDAC2, HDAC6) (Figure 2I, Supplementary Table 3 and Supplementary File 2), suggesting that the co-regulatory axis between WRN and the NMNATs are important for chromatin stability and DNA maintenance. Combined, these results suggest that loss of WRN results in dysregulation of the NAD^+^ machinery, which likely affects transcriptional regulation.

### Mitochondrial NAD^+^ is decreased in *WRN*-KD cells

**Figure 3:**
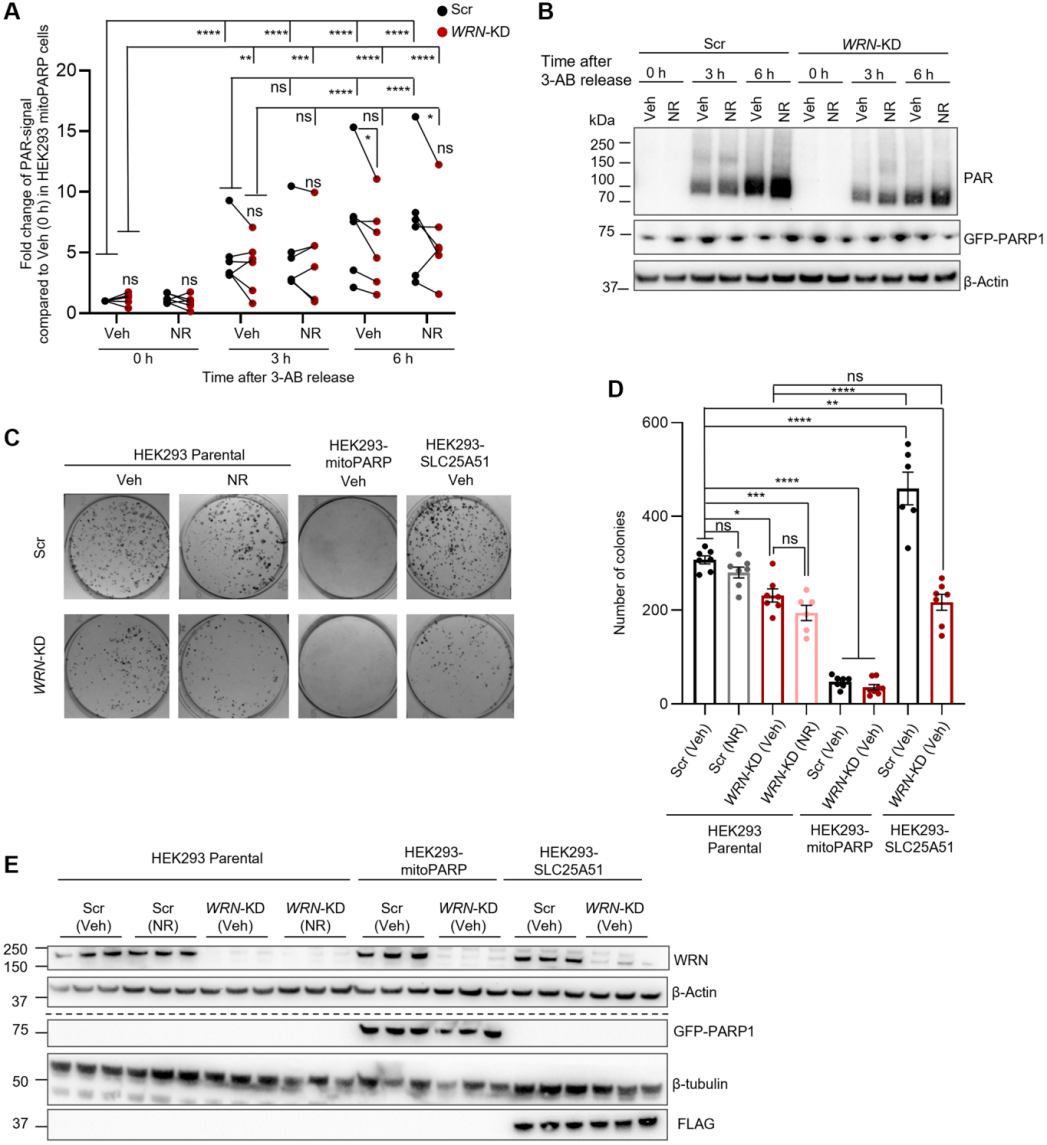
**Reduced mitochondrial NAD^+^ in WRN depleted cells.** (**A**, **B**) *WRN*-KD in the HEK293-mitoPARP reporter cell line led to decreased mitochondrial NAD^+^ compared to Scr as measured by PAR signal relative to β-Actin. Cells were grown in the presence of the PARP inhibitor 3-AB (1 mM) and collected at the designated timepoints after removal of 3-AB from the media (0, 3, 6 h). (**A**) Quantification of 6 biological repeats. Scramble (Scr) is paired with *WRN*-KD from the same biological repeat with a black line in the graph. Statistical significance was found between 0 h (right after the release of 3-AB treatment) compared to 3 h and 6 h (Two-way ANOVA, Tukey’s multiple comparisons test. Scr (Veh): 0 h to 3 h and 6 h *p*-values < 0.0001. *WRN*-KD (Veh): 0 h to 3 h *p*-value = 0.001; 0 h to 6 h *p*-value < 0.0001). Further increased PARylation signal, resembling increased mitochondrial NAD^+^ levels, was observed in Scr between 3 h and 6 h after 3-AB release (Two-way ANOVA, Tukey’s multiple comparisons, *p*-value = 0.0030), however no change was observed in *WRN*-KD cells between 3 h and 6 h, suggesting less available mitochondrial NAD^+^ (Two-way ANOVA, Tukey’s multiple comparisons, *p*-value = 0.8310). Moreover, there was a significant reduced level of PAR in *WRN*-KD cells compared to Scr at 6 h after 3-AB release (Two-ANOVA, Tukey’s multiple comparisons, *p*-value = 0.0321). (**B**) Representative western blot of PAR and β-Actin signal. (**C**, **D**) *WRN*-KD leads to decreased colony formation in HEK293 cells (Two-way ANOVA, Tukey’s multiple comparisons, *p*-value = 0.012), which was not rescued by overexpression of the human mitochondrial NAD^+^ transporter SLC25A51 or 1 mM NR treatment for 24 h. Overexpression of SLC25A51 in HEK293 (HEK293-SLC25A51) did on the other hand significantly increase colony formation in Scr cells (Two-way ANOVA, Tukey’s multiple comparisons, *p*-value = 0.0049). Colony formation assay was performed for Scr or *WRN*-KD (30 nM siRNA) in three different HEK293 cell lines: Parental HEK293, HEK293-mitoPARP and HEK293-SLC25A51. The HEK293-mitoPARP cells were exposed to 6 h 3-AB release before seeding out for colonies in medium containing 1 mM 3-AB for 11 days. (**C**) Representative images of stained colonies. (**D**) quantification of 3 biological replicates with 3 technical repeats. (**E**) Confirmation of *WRN*-KD was shown to be 90–100% in all biological replicates. Western blotting of GFP-PARP1 and FLAG confirmed the specific expression of the mitoPARP and SLC25A51-constructs, respectively. The dotted line indicates that the representative blots are from two different blots with their respective loading controls. Statistics were performed with GraphPad Prism using Two-way ANOVA with Tukey’s multiple comparison.

Due to the severe mitochondria-related dysfunctions seen in WS [6], we examined the effect of loss of WRN on mitochondrial NAD^+^. Mitochondrial NAD^+^ was measured indirectly by using HEK293 cells stably expressing mito-EGFP-PARP1cd-myc, a mitochondrially localized PARP1 protein (termed mitoPARP cells onwards) [19]. The mitoPARP cells overexpress PARP1 fused with a mitochondrial localization signal (MLS), resulting in localization of MLS-PARP1 within the mitochondria [18, 19]. Based on the function of PARP1 to construct chains of poly-ADP-ribose (PAR) by use of NAD^+^, the level of PARylation in the mitoPARP cells correlates with the amount of freely available NAD^+^, as reported and validated previously [19, 20]. Therefore, the amount of PAR signal in the mitoPARP cells can be used as a reporter of mitochondrial NAD^+^ [19, 20]. We silenced *WRN* with siRNA (*WRN*-KD) in the mitoPARP cells and treated them with either Veh or 1 mM NR for 24 h in the presence of the PARP inhibitor 3-aminobenzamide (3-AB) to avoid mitochondrial NAD^+^ depletion by the mitoPARP overexpression [19]. To measure NAD^+^ levels, we released the cells from the PARP inhibitor and collected samples at timepoints 0 h, 3 h or 6 h following release (Figure 3A, 3B). We observed a significant increase of PARylation when comparing 0 h to 3 h as expected, with no difference between Scr and *WRN*-KD cells. Six hours after 3-AB release, we saw a significant increase of PAR-signal in Scr compared to the 3 h timepoint, but no further change in the PAR-signal in *WRN-*KD cells from 3 h to 6 h (Figure 3A, 3B). Moreover, there was statistically significant less PAR-signal in *WRN*-KD cells at 6 h post 3-AB compared to Scr. This indicates that *WRN*-KD resulted in decreased mitochondrial NAD^+^ compared to Scr cells. Due to the role of WRN in DNA repair, total cellular PAR levels, have previously been shown to increase upon WRN loss, which we also observed in the parental HEK293 cells 6 h post 3-AB release (Supplementary Figure 2). We did not observe an increase in PAR-signal in either Scr or *WRN*-KD cells after 24 h with 1 mM NR treatment (Figure 3A, 3B).

### NR treatment or overexpression of SLC25A51 did not restore cell proliferation in WRN depleted immortalized cells

In the center of WS disease etiology is loss of cell proliferation and premature senescence. We have previously shown that treatment with the NAD^+^ precursors NR or NMN could rescue stress-induced intestinal stem cell proliferation in a *Drosophila melanogaster* model of WS [6]. Combined with our findings on decreased senescence markers post NR treatment in *WRN*^−/−^ MSCs and decreased mitochondrial NAD^+^ in *WRN*-KD cells, we examined the link between mitochondrial NAD^+^ and cell proliferation in *WRN*-KD cells. To increase mitochondrial NAD^+^ levels, we overexpressed SLC25A51, which has recently been identified as an NAD^+^ transporter localized in the mitochondrial membrane [20]. First, we used primary fibroblasts from WS patients as well as sex- and age-matched controls; however, we were unable to successfully transfect the SLC25A51 plasmid into the WS fibroblasts, possibly due to high vulnerability (not shown).

We then used HEK293 cells to measure proliferation via a classic colony-formation assay (Figure 3C–3E). Cells were seeded on Poly(ethyleneimine) solution coated 6-well plates and left undisturbed for 11 days, whereafter the colonies were fixed, stained, and imaged. As seen in Figure 3E, knock down of *WRN* was confirmed by western blotting. In the parental HEK293 cells, *WRN*-KD led to decreased colony formation compared to Scr, as expected (One-way ANOVA, Tukey’s multiple comparisons test, *p*-value = 0.0189). A 24 h treatment with 1 mM NR prior to colony formation assay did not affect the proliferation capacity of either Scr or *WRN*-KD cells in the parental HEK293 cells (Figure 3C, 3D). Next, we used two additional cell lines: one cell-line overexpressing the mitochondrial NAD^+^ transporter SLC25A51 (SLC25a51-myc-FLAG, called SLC25A51 from now on) [20] in order to shift the equilibrium in the cells towards increased mitochondrial NAD^+^, and in addition the mitoPARP cells described above. After 6 h of growth without 1 mM 3-AB to deplete the cells from mitochondrial NAD^+^, the mitoPARP cells were seeded for colony formation in the presence of 3-AB to avoid further depletion of mitochondrial NAD^+^. The mitoPARP cells showed an almost complete disruption of colony formation in both Scr and *WRN*-KD cells with no statistical difference (Figure 3C, 3D). It is worth noting that the continued treatment with 3-AB, will inhibit all cellular PARP activity, which likely resulted in disrupted DNA damage response and hereby cell death.

Interestingly, overexpression of SLC25A51 led to increased colony formation in Scr cells compared to the parental HEK293 Scr cells (One-way ANOVA, Tukey’s multiple comparisons test, *p*-value = <0.0001). However, no statistically significant change was observed in the parental HEK293 with *WRN-*KD compared to SLC25A51 cells with *WRN*-KD. These results indicate that the proliferation defects observed in *WRN*-KD immortalized cells cannot be rescued by increasing cellular NAD^+^ (with NR treatment) or by increasing the potential import of NAD^+^ into the mitochondria (by overexpressing SLC25A51), however cell proliferation was completely abolished when mitochondrial NAD^+^ was depleted.

### SLC25A51 overexpression combined with NR treatment does not rescue proliferation in *WRN-*KD cells

**Figure 4:**
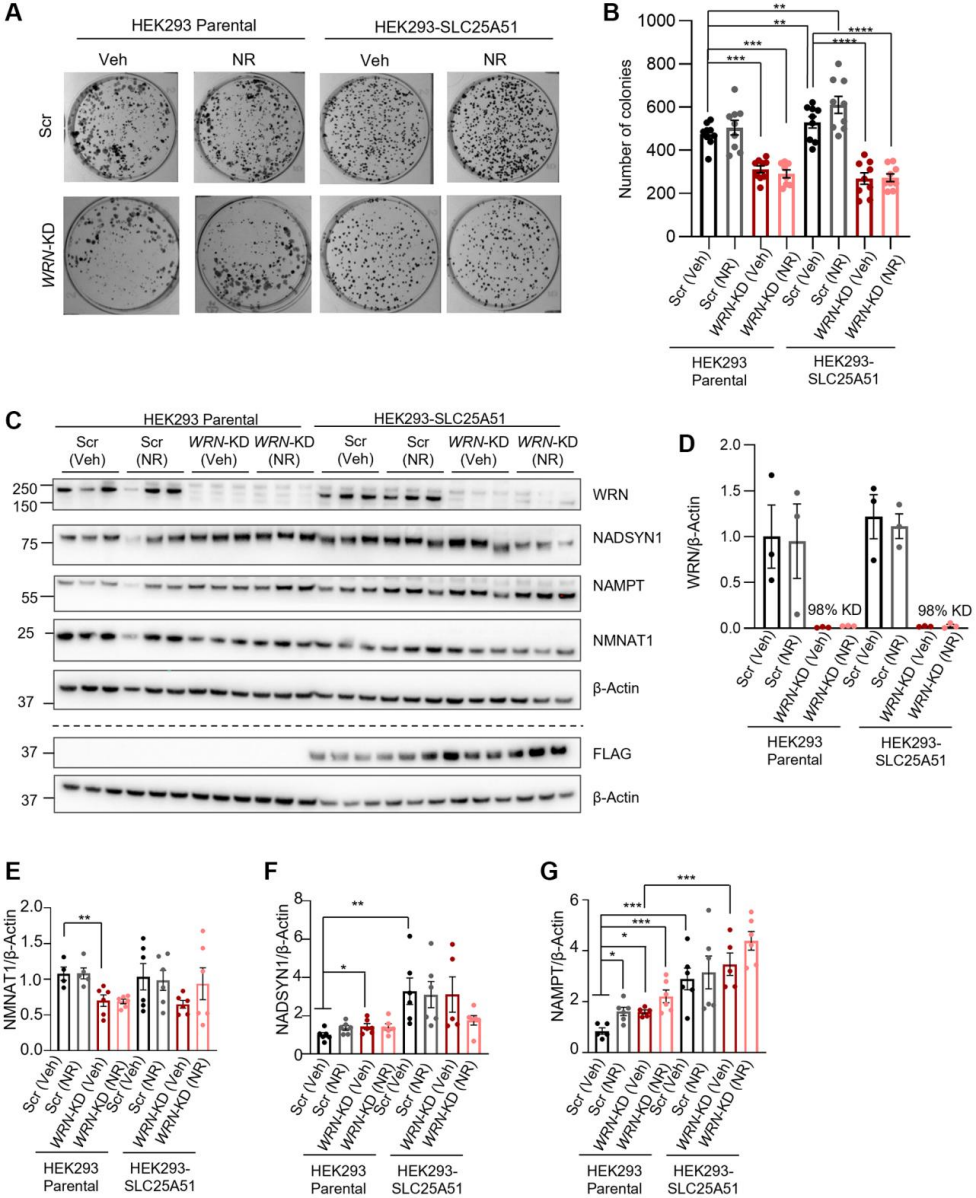
**WRN is indispensable in proliferation as mitochondrial or cellular NAD^+^ augmentation is unable to reinstall compromised proliferation in immortalized *WRN*-KD cells.** (**A**, **B**) *WRN*-KD led to decreased colony formation in HEK293 parental cells (Two-way ANOVA, Tukey’s multiple comparisons, *p*-value = 0.0009). The proliferation defects caused by knockdown were not rescued by either 1 mM NR treatment throughout the colony formation period or overexpression of SLC25A51. Media was replaced with fresh media every 3 days. (**A**) Representative images, acquired with Bio-Rad Chemidoc. (**B**) Quantification of 3 biological replicates with 3 technical repeats/replicates. Statistical analysis was done using two-way ANOVA with Tukey’s multiple comparisons test. (**C**) Western blotting confirming *WRN*-KD of 90–100% and blotting of NAD^+^ related proteins. Western blotting of FLAG confirmed the specific expression of the SLC25A51-construct. The dotted line indicates that the representative blots are from two different blots with their respective loading controls. (**D**) Quantification of WRN expression, confirming efficient knockdown of *WRN* in both HEK293 and SLC25A51. (**E**–**G**) Quantification of NAD^+^ related proteins. The groups were compared with Two-way ANOVA using Tukey’s multiple comparison to test the difference among all groups, and Student’s *t*-test as explained in Figure 2. NADSYN1 expression was increased in Scr cells with SLC25A51 overexpression compared to parental HEK293 cells (Two-way ANOVA, Tukey’s multiple comparisons test, *p*-value = 0.0048), and NAMPT was increased in both Scr and *WRN*-KD cells with SLC25A51 overexpression (Two-way ANOVA, Tukey’s multiple comparisons test, *p*-value < 0.0001 and *p*-value = 0.0004, respectively). No significant effects were found with 24 h 1 mM NR treatment.

To further understand the effect of mitochondrial NAD^+^ on proliferation, we treated parental HEK293 cells and SLC25A51-overexpressing HEK293 cells with 1 mM NR for 11 days during colony formation. The cell culture medium was exchanged every 3 days with fresh medium with or without 1 mM NR (Figure 4A, 4B). Silencing *WRN* with siRNA led to decreased colony formation capacity in both the parental and SLC25A51 HEK293 cells (One-way ANOVA, Tukey’s multiple comparisons test, *p*-value = 0.0009 and *p*-value < 0.0001, respectively). Colony formation was increased with SLC25A51 overexpression without *WRN* silencing, but not in cells with *WRN* silencing. Eleven days of 1 mM NR treatment did not affect colony formation in either Scr or *WRN*-KD cells. This suggests that mitochondrial NAD^+^ is not sufficient to rescue proliferation in *WRN* depleted HEK293 cells.

We further evaluated the effect of both NR treatment and SLC25A51 overexpression on the protein levels of NAD^+^ metabolism-related proteins by treating parental HEK293 (HEK293P) cells (same blots as in Figure 2) or SLC25A51 overexpressing cells with 1 mM NR treatment for 24 h. The results showed that neither NR nor SLC25A51 overexpression affected the expression of NMNAT1 when compared to HEK293 Parental in either Scr or *WRN*-KD cells (Figure 4C–4E). NADSYN1 expression was increased in Scr cells with SLC25A51 overexpression compared to parental HEK293 cells, but not in *WRN*-KD cells (Figure 4C, 4F; One-way ANOVA, Tukey’s multiple comparisons test, *p*-value = 0.0048)), and NAMPT was increased in both Scr and *WRN*-KD cells with SLC25A51 overexpression (Figure 4C, 4G, One-way ANOVA, Tukey’s multiple comparisons test, *p*-value < 0.0001 and *p*-value = 0.0004, respectively). No significant effects were found with 24 h 1 mM NR treatment in any of the cell lines tested.

## DISCUSSION

Patients suffering from WS display several metabolic impairments suggesting compromised cellular metabolism [3, 32]. By analyzing RNA sequencing data from human isogenic MSCs with or without *WRN*^−/−^ [5], we showed that both proliferation and cellular metabolism were altered with loss of *WRN*, and that multiple pathways related to proliferation and metabolism were restored in *WRN*^−/−^ cells when treated with NR for 24 h. When correlating the expression of a selection of genes related to cell proliferation to genes related to NAD^+^ metabolism, we observed a positive correlation between various NAD^+^ related genes, and a negative correlation between NAD^+^ related genes and senescence/proliferation stalling factors, indicating a role of NAD^+^ metabolism in the impaired proliferation seen in WS cells. We confirmed that NAD^+^ augmentation decreased senescence and increased proliferation of WS MSCs and fibroblasts, supporting the ability of NR to reduce senescence [33].

Therefore, we aimed to study the relation between NAD^+^ and proliferation in *WRN* deficient cells. Our results demonstrated that knockdown of *WRN* in HEK293 cells led to decreased proliferation measured with colony formation, enabling us to study the effects of WRN on proliferation in these cells. We also showed that mitochondrial NAD^+^ was decreased in *WRN*-KD cells compared to Scr. In accord, NMNAT1, converting NMN to NAD^+^, were decreased in *WRN*-KD cells. Twenty-four hours of treatment with 1 mM NR did not affect the mitochondrial NAD^+^ level or the expression levels of NMNAT1. Finally, we observed that overexpression of SLC25A51, a newly identified mitochondrial NAD^+^ transporter, increased proliferation of Scr cells, but not *WRN*-KD cells. Additionally, SLC25A51 overexpression led to increased expression of NADSYN1 in Scr and increased expression of NAMPT in both Scr and *WRN*-KD cells.

Mitochondrial NAD^+^ levels have previously been suggested to be critical for cellular survival during genotoxic stress. Moreover, it has been indicated that NAMPT, the rate-limiting enzyme in the NAD^+^ salvage pathway, is a stress- and nutrient-responsive gene, which boosts mitochondrial NAD^+^ [34]. In our study, we observed an increased level of NAMPT in HEK293 cells with *WRN* knockdown and simultaneously a decreased level of NMNAT1, which has also previously been shown in primary fibroblasts with *WRN*-KD [6]. This suggested an activation of NAMPT due to *WRN*-loss-induced genomic stress. Using cells overexpressing a mitochondrial-targeted PARP protein (mitoPARP), we measured PARylation as an indirect measure of mitochondrial NAD^+^, and showed that mitochondrial NAD^+^ was lower in cells with* WRN*-KD compared to Scr. Our findings indicate an impairment of mitochondrial NAD^+^ in addition to cellular NAD^+^ metabolism [6]. Future studies will evolve even more precise measures of subcellular levels of NAD^+^ [35, 36], which will enable a more detailed overview of the (im)balance of subcellular NAD^+^ pools upon loss of WRN.

Combined, our results indicate disrupted NAD^+^ metabolism in *WRN* depleted cells, and together with decreased mitochondrial NAD^+^, it points to WRN loss causing dysregulation of NAD^+^ synthesis not only in the nucleus but also in mitochondria. Unfortunately, due to the lack of reliable antibodies against NMNAT2 and NMNAT3, we were unable to evaluate the protein levels of those in our study. WRN and the NMNATs share multiple downstream transcriptional regulators pointing towards a shared transcriptional regulatory axis. Further studies are needed to clarify the regulatory link between WRN and the NMNATs, and how this potentially regulates NAD^+^ metabolism.

In this study, we observed a reduction in senescence markers following NR treatment in both *WRN*^−/−^ MSCs, and in primary fibroblasts derived from WS patients. However, we did not observe a benefit of either NR treatment or SLC25A51 overexpression in HEK293 cells with knockdown of *WRN* in terms of mitochondrial NAD^+^, measured as PARylation in mitoPARP cells, or on colony formation. On the other hand, we did observe increased proliferation of Scr HEK293. Recent studies on the role of SLC25A51 in cancers, show increased levels of SLC25A51 in multiple cancers, and furthermore loss of SLC25A51 decreased proliferation and apoptosis in two different cancer cell models [37, 38]. It was also suggested that increased SLC25A51 expression decouples mitochondrial NAD^+^/NADH from proliferation [38]. The lack of increased proliferation after SLC25A51 overexpression in *WRN* deficient cells is therefore interesting. It might be due to the use of an immortalized cell line with cancerous origin, which may again affect the cellular metabolism and mitochondrial NAD^+^ dependence for proliferation. Another explanation is the primary role of WRN as a chromatin remodeler and DNA repair protein; suggesting that perhaps the main benefits of NAD^+^ augmentation is centered in the nucleus. It has previously been shown that loss of SLC25A51 increases nuclear NAD^+^-dependent PARP1-mediated DNA repair [39]. Overexpression of SLC25A51 in *WRN* deficient cells might therefore increase mitochondrial NAD^+^, but simultaneously decrease the nuclear levels of NAD^+^, resulting in lack of proliferative-related benefits. This hypothesis is supported by the effects of NR treatment on proliferation-related pathways and the decrease of NMNAT1, the nuclear NMNAT, in *WRN* depleted cells. Further studies, such as single-cell OMICS based approaches, clarification of the change of the other NMNATs, and subcellular NAD^+^ measures [35, 36, 40, 41], are needed in order to clarify the cell-type and cell-location specific effects of both *WRN* deficiency and the impact on NAD^+^ metabolism.

There are limitations of this study, and our findings should be interpreted in the context of such limitations. Due to the high vulnerability of WS fibroblasts used, we encountered challenges including slow cell proliferation and high transfection-induced cell death, which stopped us to explore those cells further. Since the HEK293 cells are immortalized, this could minimize the therapeutic potential of NAD^+^ on impaired cellular proliferation. Indeed, this limitation should be considered when comparing the data of the benefits of NAD^+^ in maintaining ‘youth’ in human WS MSCs and primary fibroblasts (this study) and in restoring the proliferation of intestinal stem cells after gut damage in WS *Drosophila* [6]. Finally, further mechanistic studies on ‘how mitochondrial NAD^+^ is reduced’ and ‘how NAD^+^ maintains ‘youth’ of WS MSCs’ are needed.

In conclusion, NAD^+^ metabolism has been shown critical for WS models’ lifespan and health span, and NAD^+^ augmentation of WS animal models restored lifespan to that of WT level [4, 6]. This work consolidates the importance of NAD^+^ in WS and aging, but also suggests that NAD^+^ alone might not be sufficient to treat all hallmarks of WS. Future studies are needed to understand the subcellular regulation of NAD^+^ metabolism *in vivo* and in primary cells.

## MATERIALS AND METHODS

### Mesenchymal stem cells

The mesenchymal stem cells (MSCs) were generated in the laboratory of H.H. Cheung as previously published [9]. Control MSCs (WT; GM440, BM-MSCs) and WS MSCs (WS797; WS780) were seeded on 24-well plates. The day after seeding, NR treatment (0.5 mM or 1 mM) was initiated, and media was exchanged with fresh media without or with NR every third day. After 11 days (WS797), 18 days (WS780) or 24 days (GM440; BM-MSCs) cells were stained with β-Gal, and then imaged on a bright field microscope. The number of β-Gal positive cells compared to the total number of cells was quantified using ImageJ on images from two biological repeats per cell line.

### Primary fibroblasts

The primary skin fibroblasts (NF1, NF2, WFL8, WF9A) were obtained from two healthy individuals and two WS patients. Written informed consent was obtained from the individuals who provided the samples to conduct and publish this study, which was approved by the Institutional Ethics Review Board of the Chiba University Graduate School of Medicine (IRB-1145[1029]). The primary fibroblasts (AG10803A; AG16146; AG02602; AG12975; AG06300) were obtained from Coriell Institute (USA). Two WS patient derived cell lines (AG12975; AG06300) and three control lines from healthy sex- and age-matched controls (AG10803A; AG16146; AG02602) were used. All experiments with primary fibroblasts were performed in passage 14–16, and the same passages were used for both controls and WS lines. The cells were grown in DMEM/MEM (50:50) with 15% fetal bovine serum (FBS) and 1% penicillin-streptomycin (PS). For β-Gal evaluation, cells were grown without or with 1 mM NR in the medium for 10 days in total, with the media exchanged with fresh media every other day. SA-β-gal staining was performed using the Senescence β-Galactosidase Activity Assay Kit (Fluorescence, Plate-Based; #23833, Cell Signaling Technology) according to the manufacturer’s protocol. Cells cultured in 12-well plates were fixed with 1× fixative solution and stained overnight at 37°C. The cells were washed with PBS (−), stained with Hoechst 33342 (H342; Dojindo, Kumamoto, Japan) according to the manufacturer’s protocol, and photographed using a BZ-X700 microscope (Keyence, Osaka, Japan). For SA-β-Gal detection with Spider β-Gal stain (Dojindo), cells were seeded on poly-D-lysine coated coverslips. The next day the media was exchanged to media without or with 1 mM NR. The following day, Spider β-Gal stain (Dojindo) was prepared in DMSO according to the manufacturer’s protocol, then diluted in Mcllvaine buffer (pH 6.0) (0.1 mol/L citric acid solution, 0.2 mol/L sodium phosphate solution, adjusted to pH 6.0, and diluted 5 times in ultrapure water). The cells were fixed in 4% paraformaldehyde (PFA), stained for 30 min, washed in PBS, and mounted with ProLong DAPI mounting media (Invitrogen, USA). Cells were imaged with Zeiss LSM780 confocal microscope (DAPI (Ex. 405 nm), Spider β-Gal (Ex. 488 nm)) and the intensity of the Spider β-Gal signal was analyzed with ImageJ (four biological repeats, with two controls and two WS cell lines).

To evaluate senescence, the cellular localization of HMGB1, was used; decreased nuclear HMGB1, is a marker of increased senescence. Primary fibroblasts from either controls or WS patients were grown for 7 days in cell culture media without or with 1 mM NR with the media exchanged with fresh media every other day. Then, WT or WS-patient derived fibroblasts were seeded on PDL coated coverslips in normal media. The next day, media was changed to Veh or 1 mM NR. After additional 24 h, cells were washed in PBS, fixed in 4% PFA and permeabilized, then stained with anti-HMGB1 antibody (1:400, Abcam, #18256) and secondary anti-rabbit (1:500; Alexa-flour 546, Invitrogen). The cells were washed in PBS and mounted with Prolong DAPI mounting media (Invitrogen, USA). Cells were imaged with Zeiss LSM780 confocal microscope and cells expressing HMGB1 only in the nucleus compared to in the entire cell were analyzed with ImageJ (three biological repeats with three controls and two WS cell lines).

### Cell lines, cell culture, and RNAi knockdown

The HEK293, stably transfected HEK293 cell lines (mito-EGFP-PARP1cd-myc (mitoPARP), and SLC25a51-myc-FLAG (SLC25A51)) were generated in the M. Ziegler laboratory as described previously [19, 20]. Cells were grown in DMEM with 10% FBS, 1% PS and 550 µg/ml Geneticin-418. For mitoPARP cells, 1 mM 3-aminobenzamide (3-AB) was included in the medium unless otherwise noted.

To induce knockdown of *WRN*, cells were treated with 30 nM *WRN* siRNA oligo duplex for 6 h (#SR322215, OriGene) in OPTI-MEM media, followed by addition of normal growth media. After 48 h cells were collected in (Cell Signaling, #9806S) containing 1x Halts protease and phosphatase inhibitor cocktail (Thermo Fisher, USA) and the knockdown efficiency was confirmed with western blotting as described below.

### Poly-ADP-ribose assisted detection of mitochondrial NAD^+^

MitoPARP cells were seeded on poly-D-lysine (PDL) coated plates in normal growth media minus antibiotics. After 16 h, the media was replaced with OPTI-MEM media and cells were transfected with either scramble siRNA (Scr) or 30 nM WRN siRNA (OriGene, USA). After 24 h, the medium was replaced with normal growth medium +/− 1 mM NR for 24 h. In all instances the mitoPARP cell line was grown with 1 mM 3-AB in the media to avoid mitochondrial NAD^+^ depletion. To evaluate the level of mitoPARP in Scr vs. *WRN*-KD cells, the medium was replaced with medium without 3-AB, and cells were harvested at different timepoints as indicated in the figure (0 h, 3 h or 6 h after release from 3-AB). To harvest the cells, they were first washed once in 1 mL PBS, then lysed (20 mM TrisHCl (pH 7.4), 150 mM NaCl, 1% (v/v) SDS, 1 mM EDTA, 1 mM 3-AB) and collected. After passaging the lysate 10 times through a 23-gauge needle to shear the genomic DNA, protein concentration was determined using Pierce BCA protein assay kit (Thermo Fisher, #23225). Fifteen µg of lysate was separated by SDS-PAGE in a 4–12% bis-tris gel and subjected to immunoblotting using overnight incubation at 4^ο^C with primary antibodies (anti-PAR, Novus #4355-MC-100, anti-β-Actin, Sigma, #SAB1305546) followed incubation with HRP-conjugated goat anti-mouse secondary antibody for 1 h at room temperature. HRP-detection was performed using SuperSignal West Femto Maximum Sensitivity Substrate (Thermo Fisher, #34096) and ChemiDoc XRS+ imaging system (Bio-Rad, USA). The PAR signal was quantified using ImageJ.

### Colony formation assay

To evaluate proliferation capacity of *WRN*-KD cells when changing the mitochondrial NAD^+^ availability, we performed a colony formation assay. Two-hundred fifty cells were seeded in 6-well plates including 3-AB for mitoPARP cells. The next day, cells were transfected with scramble or *WRN* siRNA as described above. The following day, the medium was changed to normal DMEM +/− 1 mM NR. After 24 h, cells were trypsinized, counted, and re-seeded in coated 6-well plates for colony formation. 750 cells were seeded per well. In the experiment with continued NR treatment, the media was replaced with fresh media every three days.

On day 11, wells were washed in 1 mL PBS, fixed with ethanol and stained with 0.3% crystal violet (Sigma-Aldrich) in 70% ethanol for 30 min at room temperature following additional washes before drying. Colonies were imaged on ChemiDoc XRS+ imaging system and counted using ImageJ.

### Western blotting

Western blotting was used to examine protein expression following the protocol we reported elsewhere [15, 42]. HEK293 and SLC25A51 cells were collected, and extracts prepared using 50 µl 1× RIPA buffer (Cell Signaling, #9806S) containing 1× Halts protease and phosphatase inhibitor cocktail (Thermo Fisher, USA). Proteins were separated on a 26-well 4–12% Bis-Tris gel (Invitrogen, USA) and run for 50 min at 200 V in the NuPage Midiblot Module (Invitrogen, USA). After SDS-PAGE, gels were transferred to the PuPage Midiblot transfer Module (Invitrogen, USA) at 25 V for 30 min, followed by blocking for 1 h at RT in 5% non-fat dried milk-TBST. Membranes were incubated with primary antibodies in milk-TBST ON at 4°C, and secondary antibodies in milk-TBST for 1 h at RT before detection on ChemiDoc XRS+ imaging system (Bio-Rad, USA). Quantification was performed using ImageJ. The original western blots can be found in the Supplementary Materials. Antibodies used were: β-Actin (Sigma, #SAB1305546), WRN (Santa Cruz, # sc-5629), NMNAT1 (Cell Signaling, #98354S), NAMPT (Abcam, #ab236874), NADSYN1 (Abcam, #ab171942), GFP (Roche #11814460001), FLAG (Sigma-Aldrich, #F1804-200UG), β-Tubulin (Abcam, #6046).

### Bioinformatic analysis

#### Z-score heatmap

RNA sequencing results in the form of normalized read counts (fpkm) were obtained from our previous work on human MSCs without (WT) or with knockout of *WRN* (*WRN*^−/−^)^5^. Cells were collected after 24 h without treatment (Veh) (WT and *WRN*^−/−^) or with 1 mM NR in the cell culture medium (Only *WRN*^−/−^) and RNA sequencing analysis performed as previously described (Day 1 samples in the RNA sequencing dataset). For each of the Day 1 samples, z-scores of the fpkm values were calculated for every gene of interest related to NAD^+^ metabolism and proliferation (Supplementary Table 1 for gene list; *IDO1* was excluded from the analysis as it was undetectable in any of the samples). The obtained table of z-scores was visualized using R's pheatmap package.

#### Correlogram

For each gene of interest (Supplementary Table 1), correlation of its fpkm values across all Day 1 samples with fpkm values of every other gene of interest was calculated. The corrplot R package was used for significance testing and visualization. Unweighted centroid clustering has been chosen as the clustering method for visualization, and significance calculated based on the adjusted *p*-value.

### Statistical analysis

Statistical analysis used in this study was Student’s two-tailed unpaired *t*-test for comparison between two groups, or ANOVA with multiple comparisons for comparisons among multiple groups. All data were presented as mean ± S.D. or mean ± S.E.M. as indicated, where a *p*-value < 0.05 was considered statistically significant.

### Data availability statement

The RNA sequencing data were generated in [5], and is available from Gene Expression Omnibus with the accession number: GSE248012 (https://www.ncbi.nlm.nih.gov/geo/query/acc.cgi?acc=GSE248012). The data that support the findings of this study, including data in supplementary material of this article, are available from the corresponding author upon reasonable request.

## Supplementary Materials

Supplementary Figures

Supplementary Tables

Supplementary File 1

Supplementary File 2
