# Supplementary material for: Decreased mitochondrial NAD+ in WRN deficient cells links to dysfunctional proliferation
Source: Aging (Albany NY). 2025 Apr 2;17(4):937–59. doi: 10.18632/aging.206236 (PMC12074813; doi:10.18632/aging.206236)
Supplement: Supplementary Figures [file aging-17-206236-s001.pdf]

SUPPLEMENTARY FIGURES

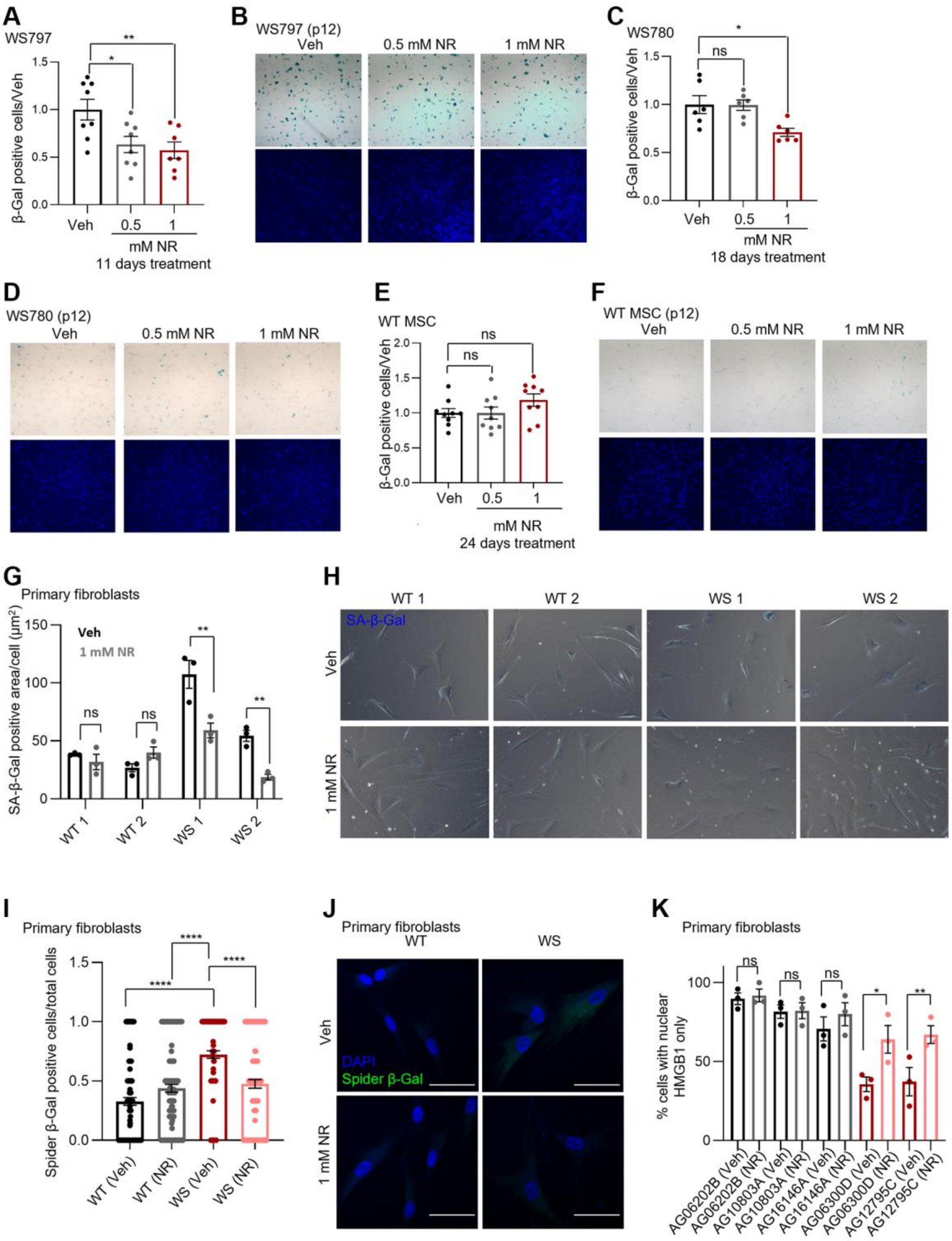

Supplementary Figure 1. NAD⁺ augmentation inhibits cellular senescence in WS MSCs and primary fibroblasts. (**A**–**F**) Quantification (**A**, **C**, **E**) and representative images (**B**, **D**, **F**) of β-Gal staining of MSCs decreased with 11–18 days of 1 mM NR treatment in WS MSCs, but no difference was seen during the similar period in WT MSCs. β-Gal positive cells relative to total number of cells were quantified from two biological experiments per cell line. (**G**, **H**) Quantification (**G**) and representative images (**H**) of SA-β-Gal staining of primary fibroblasts from two healthy donors (WT 1 and WT 2) and two WS patients (WS 1 and WS 2). Ten days treatment with 1 mM NR did not affect SA-β-Gal staining in WT 1 and WT 2, but NR treatment did significantly decrease SA-β-Gal staining in the two WS cell lines (Two-way ANOVA, Sidak's multiple comparisons, *p*-value = 0.0001 (WS 1) and 0.0030 (WS 2)). (**I**) Senescence evaluation by Spider β-Gal (Dojindo) staining of primary fibroblasts from healthy donors (WT) or WS patients (WS) without or with 1 mM NR for 10 days prior to staining. The number of cells positive for Spider β-Gal relative to the total number of cells was quantified from four biological repeats with two WT cell

lines and two WS cell lines (Two-way ANOVA, Tukey's multiple comparisons test). (**J**) Representative images of senescence evaluation by Spider β-Gal staining (Dojindo) of primary fibroblasts from healthy donors (WT) or WS patients (WS) without or with 1 mM NR for 10 days prior to staining. (**K**) Quantification of localization of HMGB1 in primary fibroblasts from healthy donors (black and grey) or WS patients (Red and pink) without (black, red) or with 1 mM NR treatment (Grey, pink) for 10 days prior to staining. The number of cells with nuclear HMGB1 only relative to the total number of cells is shown in the figure from three biological repeats from three WT cell lines and two WS cell lines (Two-way ANOVA, Sidak's multiple comparisons test). Scalebar on images 100 µm.

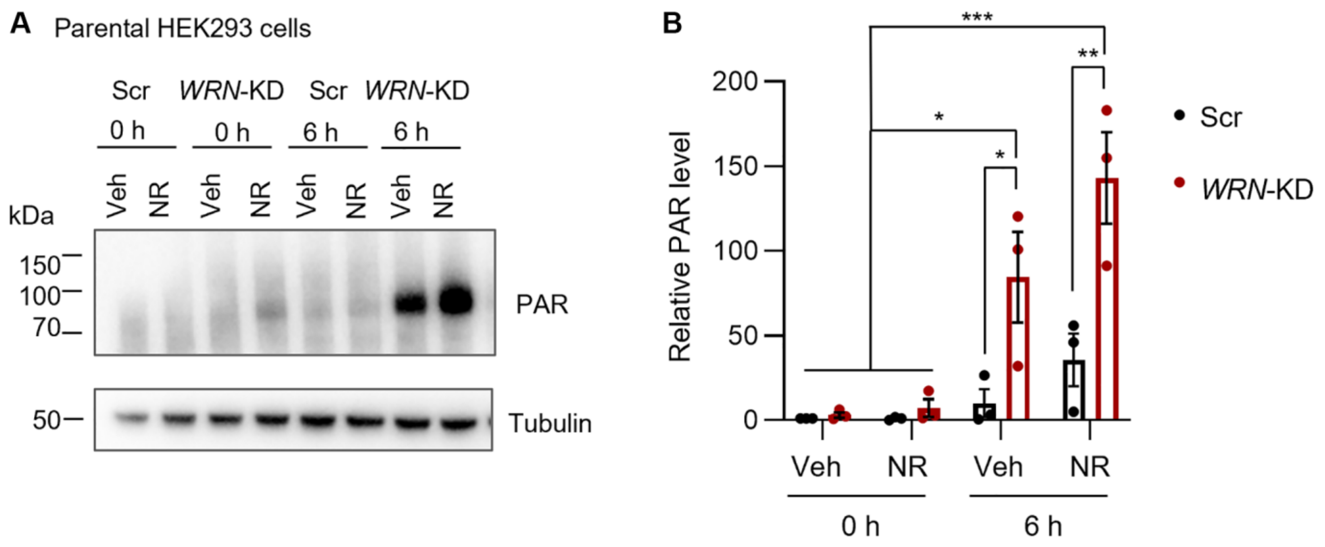

Supplementary Figure 2. Increased PARylation in *WRN*-KD HEK293 cells with/without 1 mM NR treatment compared to WT. (**A**) A set of representative Western blot images. (**B**) Quantification of PARylation level in cells with scramble siRNA or WRN siRNA +/− 1 mM NR for 24 h. Time points indicate time after release from 24 h 3-AB treatment (to resemble mitoPARP experiment in Figure 2).
