# Supplementary material for: Decreased mitochondrial NAD+ in WRN deficient cells links to dysfunctional proliferation
Source: Aging (Albany NY). 2025 Apr 2;17(4):937–59. doi: 10.18632/aging.206236 (PMC12074813; doi:10.18632/aging.206236)
Supplement: Supplementary Tables [file aging-17-206236-s002.pdf]

SUPPLEMENTARY TABLES

Supplementary Table 1. Genes of interest related to NAD+ metabolism and proliferation used for analysis of RNA sequencing results.

Gene name	Gene description
LMNB1	lamin B1 [Source:HGNC Symbol;Acc:HGNC:6637)
TP53	tumor protein p53 [Source:HGNC Symbol;Acc:HGNC:11998)
HMGB1	high mobility group box 1 [Source:HGNC Symbol;Acc:HGNC:4983]
COKN2A	cyclin dependent kinase inhibitor 2A [Source:HGNC Symbol;Acc:HGNC:1787]
COKN1A	cyclin dependent kinase inhibitor 1A [Source:HGNC Symbol;Acc:HGNC:1784]
PCNA	proliferating cell nuclear antigen [Source:HGNC Symbol;Acc:HGNC:8729)
CSF2	colony stimulating factor 2 [Source:HGNC Symbol;Acc:HGNC:2434]
MCM2	minichromosome maintenance complex component 2 [Source:HGNC Symbol;Acc:HGNC:6944]
MCM3	minichromosome maintenance complex component 3 [Source:HGNC Symbol;Acc:HGNC:6945]
NMNAT1	nicotinamide nucleotide adenylyltransferase1 [Source:HGNC Symbol;Acc:HGNC:17877]
NMNAT2	nicotinamide nucleotide adenylyltransferase2 [Source:HGNC Symbol;Acc:HGNC:16789]
NMNAT3	nicotinamide nucleotide adenylyltransferase3 [Source:HGNC Symbol;Acc:HGNC:20989]
NAMPT	nicotinamide phosphoribosy ltransferase [Source:HGNC Symbol;Acc:HGNC:30092]
NMRK1	nicotinamide riboside kinase 1 [Source:HGNC Symbol;Acc:HGNC:26057]
NMRK2	nicotinamide riboside kinase 2 [Source:HGNC Symbol;Acc:HGNC:17871]
NNMT	nicotinamide N-methyltransferase [Source:HGNC Symbol;Acc:HGNC:7861]
ID01	indoleamine2,3-dioxygenase 1 [Source:HGNC Symbol;Acc:HGNC:6059]
TD02	tryptophan 2,3-dioxygenase [Source:HGNC Symbol;Acc:HGNC:11708]
KMO	kynurenine 3-monooxygenase [Source:HGNC Symbol;Acc:HGNC:638]1
KYNU	kynureninase [Source:HGNC Symbol;Acc:HGNC:6469)
ACMSO	aminocarboxymuconate semialdehyde decarboxylase [Source:HGNC Symbol;Acc:HGNC:19288]
NAPRT	nicotinate phosphoribosyltransferase [Source:HGNCSymbol;Acc:HGNC:30450]
NAOSYN1	NAO synthetase 1 [Source:HGNC Symbol;Acc:HGNC:29832]
QPRT	quinolinate phosphoribosyltransferase[Source:HGNC Symbol;Acc:HGNC:9755]
NAOK	NAO kinase [Source:HGNC Symbol;Acc:HGNC:29831]
SARM1	sterile alpha and TIR motif containing 1 [Source:HGNC Symbol;Acc:HGNC:17074]
SIRT1	sirtuin 1 [Source:HGNC Symbol;Acc:HGNC:14929]
SIRT2	sirtuin 2 [Source:HGNC Symbol;Acc:HGNC:10886]
SIRT3	sirtuin 3 [Source:HGNC Symbol;Acc:HGNC:1493]1
SIRT4	sirtuin 4 [Source:HGNC Symbol;Acc:HGNC:14932]
SIRT5	sirtuin 5 [Source:HGNC Symbol;Acc:HGNC:14933]
SIRT6	sirtuin 6 [Source:HGNC Symbol;Acc:HGNC:14934]
SIRT7	sirtuin 7 [Source:HGNC Symbol;Acc:HGNC:14935]
COX5A	cytochrome c oxidase subunit 5A [Source:HGNC Symbol;Acc:HGNC:2267)
CYC1	cytochrome c1 [Source:HGNC Symbol;Acc:HGNC:2579]
PARP1	poly(AOP-ribose) polymerase 1 [Source:HGNC Symbol;Acc:HGNC:27]0
PARP2	poly(AOP-ribose) polymerase 2 [Source:HGNC Symbol;Acc:HGNC:272]

Supplementary Table 2. Overview of the mesenchymal stem cell lines and primary fibroblasts used in the study.

Cell line name	Genotype	Age (Years)	Sex
**Primary fibroblasts**			
AG10803A	Apparently healthy individual	22	Male
AG16146A	Apparently healthy individual	31	Male
AG026028	Apparently healthy individual	35	Female
NF1 (WT 1)	Apparently healthy individual	42	Male
FN2 (WT 2)	Apparently healthy individual	58	Male
AG06300D	Werner Syndrome	37	Male
AG12795C	Werner Syndrome	19	Male
WF8L (WS 1)	Werner Syndrome	43	Male
WF9A (WS 2)	Werner Syndrome	47	Male
**Mesenchymal stem cells**			
GM440	Control	20	Male
BM-MSC	Control	Unknown	Unknown
WS797	WRN KO (from AG12797, Coriell)	36	Male
WS780	WRN KO (from AG00780, Coriell)	60	Male

The table shows the cell line name, the genotype, age of the donors or the cells (in years), and the sex of the donors.

Supplementary Table 3. Shared transcription factor targets between WRN and NMNAT1-3 based on ChIP sequencing datasets from the ENCODE Transcription factor target database [6].

Shared targets between WRN and NMNAT1-3	
**Gene name**	**Function (GeneCards)**
BACH1	Transcription factor
BHLHE40	Control of circadian rhythm
CHD1	Chromatin regulation
CHD2	Chromatin regulation
CHO?	Helicase function, DNA binding
CTCF	Transcriptional activator or Transcriptional repressor via histone binding
E2F6	Transcription factor, cell cycle regulation
EGR1	Transcription regulation
EP300	Histone acetyltransferase
EZH2	Maintaining the transcriptional repressive state of genes over successive cell generations
GTF2F1	Chromatin regulation, RNA pol II initiation
H2AFZ	Histone family, nucleosome
HDAC2	Histone deacetylase family
HDAC6	Histone deacetylase
JUN	Transcription factor
JUND	Transcription factor
KDM4A	Histone demethylase
KDMSB	Histone demethylase
MAX	MYC associated
MAZ	Transcription factor, regulation of gene expression; regulation of signal transduction; and transcription by RNA polymerase II
MYC	Nuclear phosphoprotein that plays a role in cell cycle progression, apoptosis and cellular transformation
MYOG	Muscle-specific transcription factor
MXl1	Inhibitor of transcriptional activity of MYC by competing for MAX

PHFB	Histone lysine demethylase
POLR2A	RNA pol II
RAD21	Double-strand break repair
RBBPS	Regulates cell proliferation, histone methyltransferase
RCOR1	Interacts with REST, neuronal cell differentation
REST	Transcription factor, chromatin regulation, cancer
SAP30	Histone deacetylase, cancer
SIN3A	Transcription factor, chromatin regulation
SMC3	Chromatin regulation
TAF1	Transcription factor, RNA pol II initiation
TBP	Transcription factor
TCF12	Participate in regulating lineage-specific gene expression
TEAD4	Transcriptional enhancer
USF1	Transcription factor
ZNF143	Transcription factor, chromatin regulation
YY1	Transcription factor, chromatin regulation

Our analysis showed that WRN has 110 targets, NMNAT1 has 140 targets, NMNAT2 has 53 targets, and NMNAT3 has 74 targets (lists of all targets in Supplementary File 2). 39 targets are shared between WRN and all three NMNATs shown here.
